# Wheat Bran-Derived Zinc Phytate Mitigates Hepatic Inflammation and Metabolic Disorders Associated with Gut Microbiota-FXR–PGC-1α Signaling in High-Fat Diet-Fed C57BL/6J Mice

**DOI:** 10.3390/foods14193367

**Published:** 2025-09-29

**Authors:** Pinglian Yu, Aiqing Zhao, Mingfang Zhan, Liansheng Zhang, Chengcheng Yang, Yan Zhao, Xingbin Yang

**Affiliations:** 1 Shaanxi Key Laboratory for Hazard Factors Assessment in Processing and Storage of Agricultural Products, College of Food Engineering and Nutritional Science, Shaanxi Normal University, Xi’an 710119, China; plyu@ztu.edu.cn (P.Y.); 15218605474@163.com (M.Z.); ccyang@snnu.edu (L.Z.); chengchengya@umass.edu (C.Y.); yanzhao@snnu.com (Y.Z.); 2 School of Chemistry and Chemical Engineering, Zhaotong University, Zhaotong 657000, China

**Keywords:** wheat bran, zinc phytate, phytic acid, obesity and hepatic inflammation, gut microbiota

## Abstract

This study was designed to first investigate the effects of zinc phytate (ZnPA) from wheat bran in alleviating high-fat diet (HFD)-induced hepatic inflammation and metabolic disorders and its underlying mechanism. C57BL/6J mice were randomly assigned to five groups including normal diet (ND), HFD, HFD+low-dose ZnPA (100 mg/kg), HFD+high-dose ZnPA (200 mg/kg), and HFD+wheat bran (100 mg/kg). All interventions were administered orally for 12 weeks. The results indicated that ZnPA significantly mitigated HFD-induced weight gain, dyslipidemia, pathoglycemia, hepatic steatosis and inflammation (*p* < 0.05). ZnPA effectively corrected HFD-induced microbial dysbiosis, in which the relative abundance of the *Ruminococcus torques* group decreased from 11.0% to 0.75%, and *Coriobacteriaceae_UCG-002* dropped from 2.47% to 0.087% (*p* < 0.05). Conversely, ZnPA increased the abundance of *Ileibacterium* from 0.32% to 17.76% and *Dubosiella* from 1.03% to 4.24% (*p* < 0.05). Meanwhile, ZnPA could be metabolized by the gut microbiota to release IP6, which was further converted into secondary inositol phosphates (InsP_3–5_), resulting in increases of 52.1%, 83.3%, 62.5%, and 96.2% in the colonic contents of InsP_6_, InsP_5_, InsP_4_, and InsP_3_ (*p* < 0.05), respectively. In addition, ZnPA increased levels of secondary bile acids and short-chain fatty acids, especially deoxycholic acid and taurocholic acid, which were elevated by 1.95-fold and 1.88-fold (*p* < 0.05), respectively. Interestingly, ZnPA enhanced hepatic expressions of histone deacetylase 3, bile acid receptor FXR, and lipid metabolism coactivator PGC-1α (*p* < 0.05). Collectively, these results indicated that ZnPA might alleviate obesity-related hepatic inflammation and metabolic disorders by reshaping microbial composition and increasing the production of microbial metabolism such as secondary bile acids, thereby triggering FXR–PGC1α axis activation.

## 1. Introduction

Obesity has escalated into a serious public health concern on a global scale [1,2], and can lead to elevated levels of free fatty acids to induce insulin resistance, followed by excessive fat accumulation in the liver and a risk increase in nonalcoholic fatty liver disease (NAFLD) [3,4]. Although various therapeutic agents for NAFLD are under investigation, modification of lifestyle and physical activity by dietary changes remains the primary strategies for managing obesity and its complications [5,6]. As the “second genome” of the human body, gut microbiota can play a critical role in regulating obesity and energy metabolism homeostasis through influencing microbial communities and their metabolites [7,8,9,10,11]. However, a high-fat diet (HFD) can disrupt this balance in gut microbiota composition and bioactive metabolites, such as short-chain fatty acids (SCFAs) and bile acids [12], and notably, these alterations can be reversed through dietary interventions [13,14,15]. Thus, modulation of the gut microbiota has emerged as a potential strategy for preventing and managing obesity and related complications [16].

Zinc phytate (zinc inositol hexakisphosphate, ZnPA) is a naturally occurring complex in whole grains, legumes and oilseeds, and wheat bran (WHB) contains high levels of phytic acid and zinc, representing one of the richest natural sources of ZnPA, which is recognized for its role as a zinc supplement, targeting to the colonic microorganisms [17,18,19,20,21]. ZnPA is formed by the strong chelation between divalent zinc ions and the six phosphate groups of inositol hexakisphosphate (InsP_6_), resulting in a structurally robust complex with high chemical stability and poor solubility under physiological pH conditions [18]. This unique coordination structure imparts the substantial resistance to degradation, enabling ZnPA to retain its molecular integrity during food processing, storage and gastrointestinal digestion.

It has also been debated whether the physicochemical stability of ZnPA that underpins its persistence in food matrices might also limit its dissociation within the gastrointestinal tract, particularly under the near-neutral pH environment of the small intestine [21]. However, emerging evidences have suggested that InsP_6_ exerts diverse physiological benefits, including anti-inflammatory [22], antioxidative and anti-obesity effects [23]. Notably, both InsP_6_ and its metabolites as lower phosphorylated derivatives (e.g., InsP_3_) have been implicated in mitigating intestinal inflammation, maintaining intestinal barrier integrity and modulating gut microbiota composition via modulation of histone deacetylase 3 (HDAC3) activity, thereby contributing to gut homeostasis [24]. However, it remains unknown whether wheat bran ZnPA, which is poorly absorbed in the upper gastrointestinal tract, can be metabolized by the gut microbiota to release free InsP_6_ and zinc, thereby exerting protective effects against hepatic steatosis and liver inflammation in HFD-fed obese mice.

In this study, we explored the digestive fate and biotransformation of ZnPA extracted from WHB and its prophylactic or therapeutic effects on hepatic steatosis and inflammation in mice with obesity. Furthermore, 16S rRNA sequencing combined with non-targeted metabolomics was applied to elucidate the biotransformation of ZnPA and its underlying protective mechanisms, with the aim of clarifying how gut microbiota and their metabolites contribute to its ameliorative effects on hepatic steatosis and inflammation in mice.

## 2. Materials and Methods

### 2.1. Chemicals and Reagents

Wheat bran (WHB) was purchased from an online store (Heze City, Shandong Province, China). The normal feed (AIN-93G) and high-fat feed (AIN-93G+30%lard) were purchased from Xietong Pharmaceutical Bio-engineering Co., Ltd. (Nanjing, Jiangsu Province, China). The serum lipid metabolism factor kits and the inflammatory factor kits were obtained from Nanjing Jiancheng Bioengineering Institute (Nanjing, China). lipopolysaccharide (LPS) was supplied by Shanghai Enzyme Link Biotechnology Co., (Shanghai, China). The primary antibody against the Nuclear Receptor Subfamily 1 Group H Member 4 (NRIH4), peroxisome proliferator-activated receptor-gamma coactivator 1-alpha (PGC-1α) and Tubulin β, and horseradish peroxidase (HRP)-linked secondary antibody (goat anti-mouse or goat anti-rabbit) were purchased from Abcam (Cambridge, MA, USA). The SCFAs standards were purchased from Chengdu Munster Biotechnology Co., Ltd. (Chengdu, China). The RT-qPCR-related reagents were provided by Sangon Biotech (Shanghai, China). The standards of inositol triphosphates (InsP_6–3_) were purchased from Chengdu Cayman Chemical Co., Ltd. (Ann Arbor, MI, USA). All the other reagents and chemicals were of analytical grade or above.

### 2.2. Extraction of Zinc Phytate (ZnPA) from WHB

ZnPA was enzymatically hydrolyzed from WHB using a combination of cellulase, xylanase, pectinase, and β-glucanase at 50 °C for 3 h, under pH 5.0 and a liquid-to-solid ratio of 10:1 (*v*/*w*). The ZnPA concentration is calculated indirectly based on the content of phytate (PA). The Phytic acid content was analyzed following the National Food Safety Standard (GB 5009.153-2016, Determination of Phytic Acid in Foods, China, 2017). The concentration of ZnPA in the WHB is 120.50 ± 5.7 mg/g, and the purity of ZnPA extract is 57.3 ± 2.2% [25,26,27].

### 2.3. Animal Experiments

Male mice (C57BL/6, 4–6 weeks old) were obtained from the Experimental Animal Research Centre of Shaanxi Normal University. All animals were maintained under standard laboratory conditions in an environmentally controlled room at 22 ± 2 °C with 55–65% relative humidity and a 12 h light/dark cycle. All animal experiments were conducted in accordance with the protocols approved by the Committee on the Care and Use of Laboratory Animals of Shaanxi Normal University (C32202015).

After one week of acclimation period, C57BL/6 mice were randomly divided into five groups (*n* = 10 per group): normal diet (ND), high-fat diet (HFD), HFD+100 mg/kg ZnPA as crude extract (HFD+L-ZnPA), HFD+200 mg/kg ZnPA (HFD+H-ZnPA), and HFD+100 mg/kg WHB (HFD+WHB). To ensure reliable control, ZnPA and WHB were prepared as aqueous suspensions at the specified concentrations. Prior to dissolution, the WHB was finely ground into a powder to allow for easy aspiration using a syringe, and before each oral gavage, the suspension was thoroughly mixed to ensure uniformity, followed by oral administration. All the mice in the HFD, HFD+L-ZnPA, HFD+H-ZnPA, and HFD+WHB groups were fed with modified AIN-93G diet containing 30% lard. In addition, mice in the treatment groups were orally gavaged daily with ZnPA or WHB at doses of 100 and 200 mg/kg, which were selected based on previously published studies [23]. The mice in ND and HFD groups received an equal volume of physiological saline. Food intake (g/day) and body weight (g) were recorded weekly for each mouse. After 12 weeks, the mice were euthanized, and the liver, epididymal white adipose tissue (eWAT), inguinal white adipose tissue (iWAT), and brown adipose tissue (BAT) were quickly excised and collected for further analysis. Blood samples were centrifuged at 3000× *g* for 10 min to obtain serum, and all remaining tissues were stored at −80 °C until further analysis.

### 2.4. Oral Glucose Tolerance Test (OGTT) and Insulin Tolerance Test (ITT)

OGTT and ITT were performed as described in previously published studies [28]. Mice were administered 1.5 g/kg glucose by oral gavage or 0.75 U/kg insulin by intraperitoneal injection, and blood glucose levels were measured from tail vein blood at 0, 15, 30, 60, 90 and 120 min after glucose administration.

### 2.5. Biochemical Index Analysis of Serum and Liver

Serum levels of LDL-C, HDL-C, TG, TC, LPS, IL-1β, TNF-α, and IL-6 were determined using commercial diagnostic kits according to the manufacturers’ instructions.

### 2.6. Histological Analysis

Histological and lipid analyses of liver tissues were conducted as described in prior studies [29].

### 2.7. 16S rDNA Gene Amplification and MiSeq Sequencing

Total bacterial genomic DNA was extracted from fecal samples following previously established protocols. The V3–V4 hypervariable regions of the bacterial 16S rRNA gene were amplified by PCR. PCR products were verified by agarose gel electrophoresis, purified with a commercial kit, quantified, and pooled at equimolar concentrations for high-throughput sequencing on the Illumina MiSeq/HiSeq [30].

### 2.8. Analysis for Inositol Phosphates in Feces Samples

Inositol phosphates (InsP_3_, InsP_4_, InsP_5_, InsP_6_) were quantitatively analyzed using LC-MS/MS (AB Sciex EXION LC with QTRAP 4500). Fecal samples were extracted with 0.5 M HCl and then neutralized using a NaOH and EDTA alkaline solution. The neutralized samples were reconstituted in 1 mL of a 5:95 *v*/*v* acetonitrile–water mixture. The LC-MS/MS method was based on previously published studies [31,32].

### 2.9. Assay for Short-Chain Fatty Acids (SCFAs) in Colon Feces

The SCFA levels, including acetic, propionic, isobutyric, butyric, isovaleric, and valeric acids, in mouse colonic contents were analyzed via GC-MS. SCFAs extraction and GC-MS analysis were performed following established protocols [33].

### 2.10. Metabolomics Analysis of Feces

Metabolite profiling of mouse fecal samples was performed using UPLC-QTOF-MS/MS in both positive and negative electrospray ionization (ESI) modes. Fecal metabolites were extracted following a previously published method with minor modifications. Briefly, feces (100 mg) was mixed with 1 mL of ice-cold acetonitrile:methanol:water (1:1:1, *v*/*v*/*v*), vortexed, and subjected to ultrasonic extraction. After centrifugation at 10,000 rpm for 10 min at 4 °C, the supernatant was filtered through a 0.22 μm PVDF membrane before UPLC-QTOF-MS/MS analysis, which was conducted according to established protocols [30].

### 2.11. Quantitative RT-PCR Assay for HDAC3

Total RNA was extracted from liver and intestinal tissues using TRIzol reagent (Invitrogen, Carlsbad, CA, USA) according to the manufacturer’s instructions. cDNA was synthesized using a commercial reverse transcription kit. Quantitative real-time PCR (RT-qPCR) was performed with SYBR Green Master Mix (Sangon Biotech, Shanghai, China). Primers for HDAC3 were 5′-TGGAACAGGTGACATGTATGAA-3′ (forward) and 5′-GAAAAGGTGCTTGTAACTCTGG-3′ (reverse), and primers for β-actin (internal control) were 5′-CTACCTCATGAAGATCCTGACC-3′ (forward) and 5′-CACAGCTTCTCTTTGATGTCAC-3′ (reverse). Relative gene expression was calculated using the 2^−ΔΔCT^ method, normalized to β-actin [34].

### 2.12. Western Blot Analysis

Liver tissues were homogenized in RIPA buffer containing PMSF (100:1) and centrifuged at 12,000× *g* for 10 min at 4 °C. Protein concentrations were measured by BCA assay. An amount of 30 μg protein were separated by SDS-PAGE, transferred onto PVDF membranes, blocked, and incubated overnight at 4 °C with primary antibodies against PGC-1α (1:5000), NR1H4 (1:2000), and β-Tubulin (1:50,000). After 1 h incubation with secondary antibodies at room temperature, protein bands were visualized using enhanced chemiluminescence and quantified with Image Lab software (6.0.1) [35].

### 2.13. Data Analysis

Data are presented as the mean ± standard deviation (SD). Normality was assessed using the Shapiro–Wilk test. For normally distributed data, one-way ANOVA followed by Tukey’s post hoc test was used to evaluate group differences. Non-normally distributed data were analyzed using the Kruskal–Wallis test with Dunn’s post hoc comparisons. A *p*-value < 0.05 was considered statistically significant.

## 3. Results

### 3.1. ZnPA Decreases Body Weight and Alleviates Glucolipid Metabolism and Hepatic Steatosis in HFD-Fed Mice

**Figure 1:**
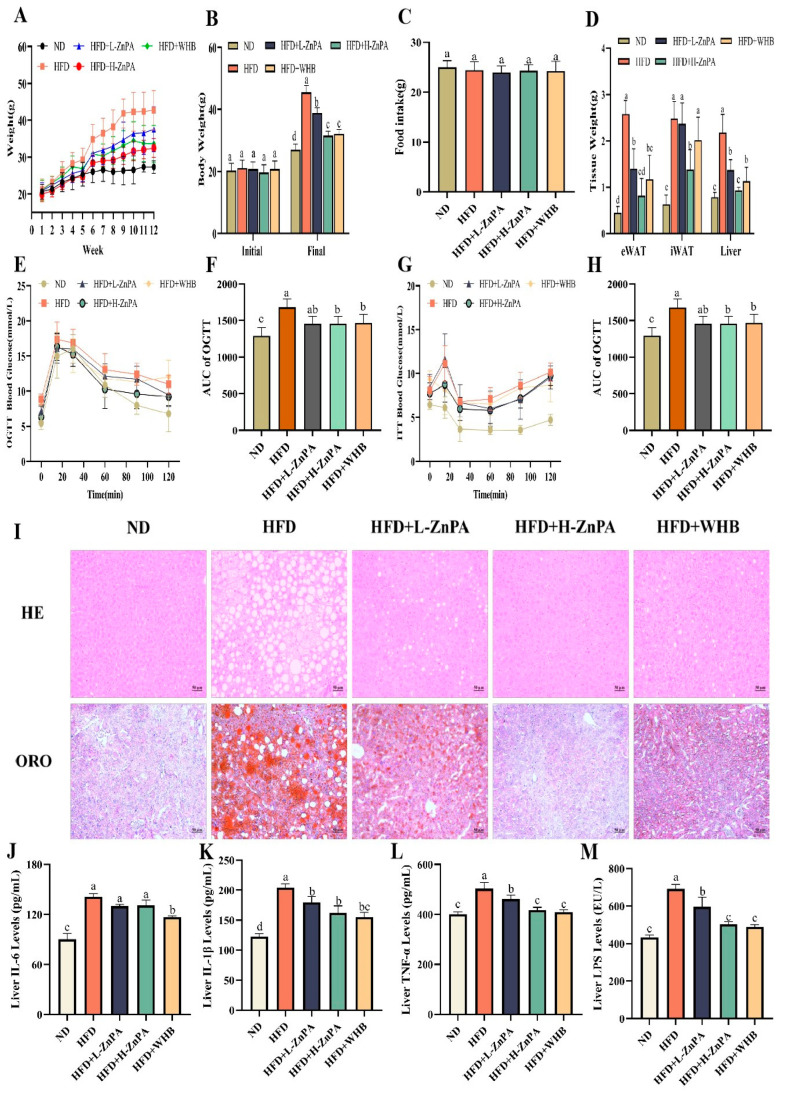
ZnPA Decreases Body Weight and Alleviates Glucolipid Metabolism and Hepatic Steatosis in HFD-Fed Mice. Body weight curves (**A**), initial and final body weight (**B**), food intake (**C**), tissue weight of the epididymal white adipose tissue (eWAT), the inguinal white adipocyte tissue (iWAT), and liver (**D**). OGTT (**E**) and AUC of OGTT (**F**) were performed by blood glucose content. ITT (**G**) and AUC of ITT (**H**) was calculated among groups is denoted by different letters. H&E and Oil Red O staining of mice liver was observed at 200× of magnification, legend: 50 μm. (**I**). Levels of inflammatory factors and LPS (**J**–**M**), in mice liver. Data are the mean ± SD (*n* = 8). Values with different letters (a, b, c, d) are significantly different (*p* < 0.05).

To clarify the effect of zinc phytate (ZnPA) from wheat bran (WHB) on high-fat diet (HFD)-induced liver injury and obesity, the experimental mice were weighed weekly before euthanasia. As expected, with an initial body weight of 21.5 ± 0.8 g, the mice in the HFD group experienced a rapid weight gain after 12 weeks of HFD feeding (Figure 1A). By the end of the period, their body weight reached approximately 45.5 ± 2.2 g, representing a 52.75% increase compared to the initial weight (*p* < 0.05). However, administration of mice with ZnPA and WHB significantly mitigated the HFD-induced weight gain in a dose-dependent manner. Specifically, at doses of 100 mg/kg and 200 mg/kg, ZnPA reduced body weight by approximately 19.07% (*p* < 0.05) and 46.98% (*p* < 0.05), compared to the HFD group, respectively (Figure 1B). In contrast, treatment of the ZnPA-enriched WHB at 100 mg/kg led to a reduction of approximately 49.77% (*p* < 0.05). Moreover, there was no significant difference in food intake of mice in the tested group (Figure 1C, *p* > 0.05). These data indicated that ZnPA and WHB supplementation exerted the inhibitory effects on body weight gain in HFD-fed mice without impact on food intake. Additionally, supplementation of ZnPA at 100 or 200 mg/kg also effectively reduced adipose tissue accumulation and liver weight. To be specific, ZnPA at 200 mg/kg significantly reduced iWAT and eWAT weights by 44.7% and 68.3%, and decreased liver weight by 27.5% relative to the HFD group, respectively (Figure 1D, *p* < 0.05).

Furthermore, administration of ZnPA significantly ameliorated glucose metabolic disturbances induced by HFD feeding in C57BL/6J mice. In comparison to the HFD group, 200 mg/kg ZnPA significantly improved glucose homeostasis, as evidenced by reductions of 28.9% and 24.3% in the AUC of OGTT and ITT, respectively (Figure 1E–H, *p* < 0.05). An amount of 100 mg/kg ZnPA also showed significant improvements, with OGTT and ITT AUCs reduction by 26.2% and 21.7%, relative to the HFD group, respectively (*p* < 0.05), although the effect was slightly less pronounced than that of the higher dose. Similarly, 100 mg/kg WHB resulted in comparable improvements in reducing OGTT and ITT AUCs by 27.6% and 23.1%, versus the HFD group, respectively (*p* < 0.05). These results indicate that all three treatments alleviated the HFD-induced glucose dysregulation to a similar extent. However, there were no significant differences in the results of OGTT and ITT between the ZnPA (both 100 and 200 mg/kg) and WHB treatment groups (*p* > 0.05), suggesting comparable efficacy in improving glucose metabolism.

Moreover, disruption of lipid metabolism is one of the core factors underlying the occurrence of liver inflammation. Herein, we systematically evaluated key blood lipid parameters. As expected, compared with the HFD group, ZnPA application showed superior efficacy in correcting dyslipidemia. At 200 mg/kg, ZnPA significantly decreased plasma TG by 33.1%, LDL-C by 40.7%, and TC by 27.8%, while increased HDL-C by 36.4% (Table 1, *p* < 0.05). Although 100 mg/kg WHB treatment produced changes in the same direction, the extent of these effects was consistently smaller, indicating a more potent lipid-regulating capacity of ZnPA. The HE staining and Oil Red O staining results of the liver further demonstrated that ZnPA markedly alleviated hepatocyte hypertrophy and lipid droplet accumulation in hepatic tissue (Figure 1I). Moreover, compared with the HFD-fed group, ZnPA at 200 mg/kg significantly suppressed hepatic inflammation, as shown by reductions in IL-6, IL-1β, TNF-α, and lipopolysaccharide (LPS) levels by 57.2%, 60.2%, 52.8%, and 34.5%, respectively (Figure 1J–M, *p* < 0.05). These anti-inflammatory effects were also observed in the WHB-treated group at 100 mg/kg. It was the first discovery that ZnPA from WHB exerted a positive improvement in hepatic steatosis and liver inflammation induced by HFD feeding.

### 3.2. ZnPA Restores Gut Microbiota Dysbiosis in HFD-Fed Obese Mice

**Figure 2:**
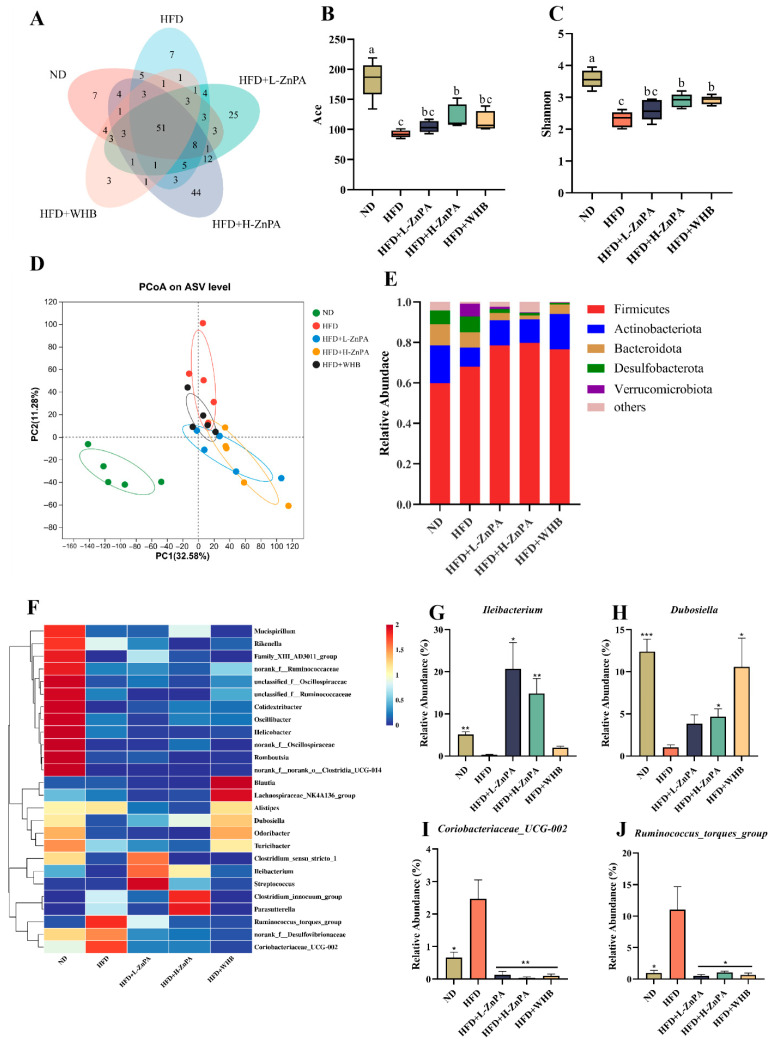
ZnPA alleviates gut microbiota imbalance in obese mice. Operational taxonomic unit (OTU) level diversity of intestinal microbiota (**A**). Analysis of intestinal microbiota richness and diversity (**B**,**C**). Community dissimilarity analysis (**D**). Microbial community distribution at the phylum level (**E**). Microbial community distribution at the genus level (**F**). Relative abundance of *Ileibacterium*, *Dubosiella*, *Ruminococcus_torques_group* and *Coriobacteriaceae_UCG-002* (**G**–**J**). Values are the mean ± standard deviation. Significance: * *p* < 0.05, ** *p* < 0.01, *** *p* < 0.001. Values with different letters (a, b, c) are significantly different (*p* < 0.05).

As shown in Figure 2A, a total of 86 operational taxonomic units (OTUs) were obtained from the feces of mice in the ND, HFD, HFD+L-ZnPA, HFD+H-ZnPA and HFD+WHB groups, of which 51 OTUs were common to the five groups. However, the richness and diversity of gut microbiota were markedly decreased in the HFD group mice compared to the ND group mice, and this reduction was inhibited by ZnPA and WHB treatment. Supplementation with ZnPA and WHB reversed the HFD-induced reduction in diversity and richness of intestinal flora. The Shannon and Simpson diversity indices point to a significant reduction in gut microbiota diversity in obese mice compared to the ND group (Figure 2B,C, *p* < 0.05). However, administration of ZnPA at 200 mg/kg led to a significant increase in these indices, restoring diversity closer to that of the ND group (*p* < 0.05). In contrast, ZnPA at 100 mg/kg did not result in a significant alteration of these diversity indices when compared to the HFD group, but the 200 mg/kg dose of ZnPA was more effective in improving gut microbiota diversity (*p* < 0.05). As shown in Figure 2D, Principal Coordinate Analysis (PCoA) further confirmed the significant differences in the composition of the gut microbiota among these five groups. Notably, the 200 mg/kg ZnPA group clustered much closer to the ND group than to the HFD group, indicating that ZnPA at 200 mg/kg was more effective in restoring microbial composition towards a state similar to that of the ND group. In contrast, ZnPA at 100 mg/kg showed a comparatively lower effect on improving the microbial composition.

The most abundant bacterial phyla were *Firmicutes*, *Actinobacteriota*, *Bacteroidetes*, and *Desulfobacterota* (Figure 2E). The abundance of *Firmicutes* was considerably increased, while the abundance of *Bacteroidetes* was significantly reduced in HFD-fed mice, relative to the ND group (Figure 2E, *p* < 0.01). Differing from the HFD group, intervention of HFD-fed mice with ZnPA resulted in an increased abundance of *Bacteroidetes* and a reduction in *Firmicutes*, indicating that ZnPA supplementation sharply regulated gut microbial composition at the phylum level.

We further identified the correlated genera in different treatment groups. From the heatmap, it can be seen that at the genus level, after supplementation with HFD, the relative abundances of the *Ruminococcus torques group*, *norank_f__Desulfovibrionaceae*, *Akkermansia*, *Coriobacteriaceae_UCG-002*, and *Clostridium innocuum group* were increased, while the abundances of *Ileibacterium* and *Dubosiella* significantly were decreased when compared to ND mice (Figure 2F), where relative to the ND group, obese mice exhibited a significant increase in the abundance of *Ileibacterium* and *Dubosiella* was significantly reduced by 7.10% and 5.35%, respectively (Figure 2G,H, *p* < 0.001), and in contrast, the relative abundance of *Coriobacteriaceae_UCG-002* and *Ruminococcus torques*, rising by 2.44% and 10.13%, respectively (Figure 2I,J, *p* < 0.001). Notably, the relative abundance of *Ruminococcus torques* was decreased from 11.0% to 0.75% (*p* < 0.001), and *Coriobacteriaceae_UCG-002* was dropped from 2.47% to 0.087% (*p* < 0.001) following ZnPA treatment. In contrast, *Ileibacterium* abundance was increased from 0.32% to 17.76% (*p* < 0.001), and *Dubosiella* abundance was elevated from 1.03% to 4.24% (*p* < 0.001) following 200 mg/kg ZnPA treatment. These findings suggest that ZnPA at 200 mg/kg provides a more substantial effect on restoring the gut microbiota diversity, enriching beneficial bacterial taxa, and reversing dysbiosis induced by HFD feeding.

### 3.3. ZnPA Regulates Metabolites of ZnPA and SCFAs Profiles in Obese Mice

**Figure 3:**
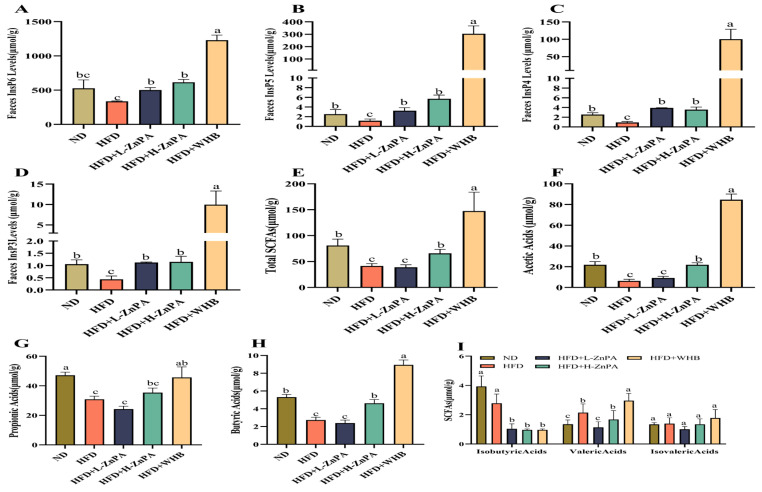
ZnPA Regulates Metabolites of ZnPA and SCFAs Profiles in Obese Mice. Inositol hexaphosphate (InsP_6_, **A**), inositol pentakisphosphate (InsP_5_, **B**), inositol tetraphosphate (InsP_4_, **C**), inositol triphosphate (InsP_3_, **D**), SCFAs (**E**–**I**) in the HFD-fed mice feces. Values with different letters (a, b, c) are significantly different (*p* < 0.05).

To evaluate whether ZnPA can release free phytic acid as inositol hexaphosphate (InsP_6_) during gastrointestinal digestion, which might further be enzymatically hydrolyzed by colon microbiota-derived phytases into inositol pentakisphosphate (InsP_5_), inositol tetraphosphate (InsP4), and inositol triphosphate (InsP_3_). In this study, it was interesting to observe that InsP_6_ remained the predominant inositol phosphate state where administration of mice with 200 mg/kg ZnPA significantly increased the levels of InsP_6_, InsP_5_, InsP_4_, and InsP_3_ by 52.1%, 83.3%, 62.5%, and 96.2%, as compared to the HFD-treated mice, respectively (Figure 3A–D, *p* < 0.05). Notably, ZnPA-treated mice displayed consistently higher inositol phosphate levels compared with the WHB group mice (100 mg/kg), suggesting that ZnPA treatment might lead to the more efficient metabolism and higher production of inositol phosphates, potentially due to the absence of degradation effects from the WHB cell wall.

Additionally, short-chain fatty acids (SCFAs) play critical roles in energy metabolism and body weight regulation by triggering various physiological responses. The treatments of ZnPA, particularly at 200 mg/kg, and WHB at 100 mg/kg significantly increased total SCFAs levels in the mouse colon when compared to the HFD-fed mice (Figure 3E, *p* < 0.05). Among the most abundant SCFAs, acetic acid, propionic acid and butyric acid exhibited a dose-dependent upregulation following ZnPA treatment (Figure 3F–H, *p* < 0.05). A similar trend was observed for valeric acid (Figure 3I, *p* < 0.05). However, both ZnPA and WHB supplementation had little effect on isobutyric acid and isovaleric acid (Figure 3I, *p* < 0.05). As a result, while both ZnPA and WHB contributed to an increase in production of SCFAs and inositol phosphate metabolites, 200 mg/kg of ZnPA appeared to be more effective than WHB and ZnPA at 100 mg/kg, highlighting the enhanced benefits at this higher ZnPA concentration.

### 3.4. ZnPA Regulates Colonic Metabolites Related to Bile Acid Metabolism in HFD-Fed Obese Mice

**Figure 4:**
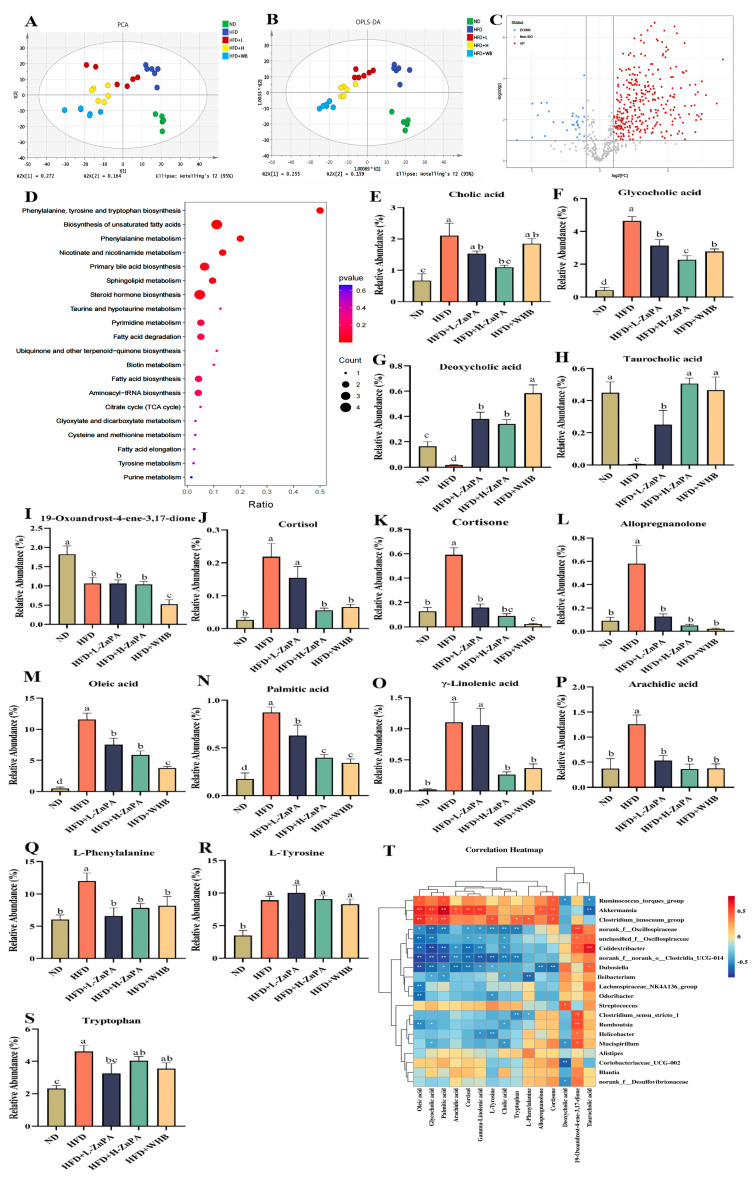
The effect of ZnPA on colonic metabolites in obese mice. PCA and OPLS-DA (**A**,**B**). Volcano plot (**C**). Pathway enrichment plot (**D**). The levels of metabolites based on the KEGG pathway analysis, including bile acids (**E**–**H**), steroid-related metabolites (**I**–**L**), fatty acids (**M**–**P**), and amino acids (**Q**–**S**). Correlation between gut microbiota and intestinal metabolites (**T**). Values with different letters (a, b, c, d) are significantly different (*p* < 0.05). * *p* < 0.05, ** *p* < 0.01.

To investigate the regulatory effects of ZnPA on colonic metabolites, a non-targeted metabolomics approach was employed. As shown in Figure 4A,B, Principal component analysis (PCA) and orthogonal partial least squares discriminant analysis (OPLS-DA) revealed the distinct clustering differences among groups, indicating that ZnPA and WHB treatments significantly altered the metabolic profiles. As depicted in Figure 4C, the volcano plot further identified several differential metabolites between the ZnPA- and HFD-treated groups. KEGG pathway enrichment analysis (Figure 4D) highlighted that ZnPA primarily affected the pathways related to bile acid biosynthesis (Figure 4E–H), steroid hormone biosynthesis (Figure 4I–L), and fatty acids metabolism (Figure 4M–P), particularly involving in phenylalanine, tyrosine, and tryptophan metabolism (Figure 4Q–S). Notably, ZnPA significantly modulated the fecal bile acid composition, which was markedly suppressed under HFD control feeding.

In comparison with the ND group, the level of deoxycholic acid (DCA) in the HFD feeding group was decreased from 1.00 to 0.40, reflecting an approximately 60% reduction. An amount of 200 mg/kg ZnPA treatment effectively restored DCA levels to 0.78, which represented an approximately 1.95-fold increase over the HFD control (*p* < 0.05, Figure 4G). The 100 mg/kg of ZnPA treatment resulted in an increase to 0.66, representing a 1.65-fold elevation, while treatment of WHB at 100 mg/kg further elevated DCA levels to 0.90, corresponding to a 2.25-fold increase (*p* < 0.05). Although WHB achieved the highest DCA level, the high-dose of ZnPA (H-ZnPA) demonstrated a substantial and consistent restoration of bile acid composition, suggesting a superior metabolic regulatory effect. Similarly, taurocholic acid (TCA) levels, which were significantly reduced in the HFD feeding from 1.00 to 0.43 (a decrease of about 57%), showed a marked recovery upon ZnPA treatment, in which H-ZnPA treatment increased TCA levels to 0.81, representing a 1.88-fold increase over the HFD group (*p* < 0.05, Figure 4H). The treatment of ZnPA at low dosage raised TCA to 0.70 as a 1.63-fold increase, while WHB treatment elevated it to 0.85, corresponding to a 1.98-fold increase (*p* < 0.05). These results indicated that 200 mg/kg ZnPA promoted a favorable modulation of bile acid levels, showing a comparable or more balanced effect than the 100 mg/kg of ZnPA and WHB treatments. Based on the above results, we speculate that ZnPA treatment may inhibit the metabolism of TCA and DCA, thereby affecting intestinal fat absorption or energy metabolism.

Further correlation analysis between gut microbiota and colonic metabolites (Figure 4T) revealed the significant associations between various bile acids and specific bacterial genera. For instance, *Akkermansia* showed a positive correlation with taurohyodeoxycholic acid, suggesting its potential role in bile acid synthesis or transformation. In contrast, the colonic *Ruminococcus_torques_group* exhibited significant negative correlations with multiple bile acids, indicating its potential sensitivity to bile acid levels. Additionally, some genera such as *Dubosiella and norank_f__Oscillospiraceae* were positively correlated with bile acids, suggesting a synergistic role in bile acid metabolism to maintain intestinal homeostasis. In summary, ZnPA effectively reversed the HFD-induced reduction in fecal bile acids, demonstrating a superior metabolic regulatory effect, which might be closely linked to bile acid metabolism and interactions with the gut microbiota.

### 3.5. ZnPA Regulates HDAC3 and FXR–PGC-1α Signaling in the Liver of Obese Mice

**Figure 5:**
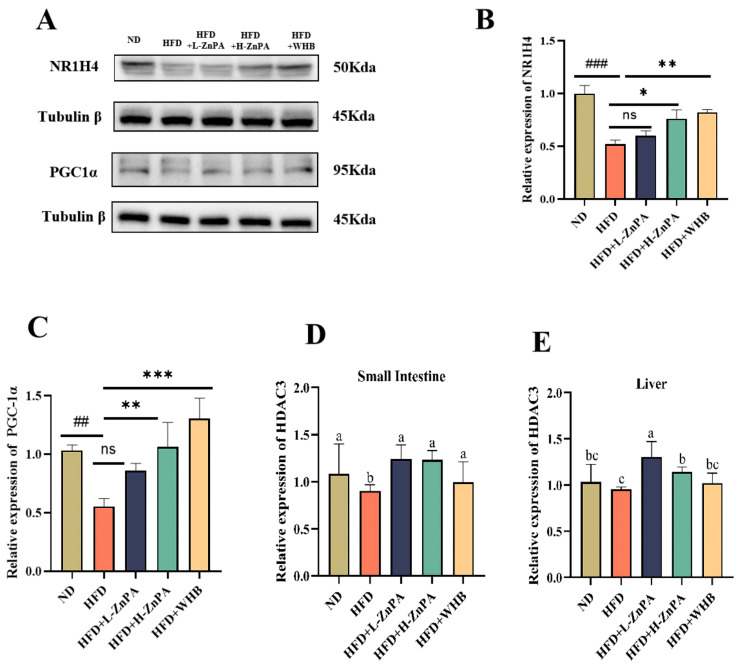
ZnPA Regulates HDAC3 and FXR–PGC-1α Signaling in the Liver of Obese Mice. Immunoblots of NR1H4 and PGC-1α in the Liver (**A**). The relative expression of NR1H4 and PGC-1α (**B**,**C**). The relative expression of HDAC3 mRNA in the small intestine (**D**) and the liver (**E**). * *p* < 0.05, ** *p* < 0.01, and *** *p* < 0.001. Values with different letters (a, b, c) are significantly different (*p* < 0.05). ^##^ *p* < 0.01, ^###^ *p* < 0.001 and ns indicates no significant difference, versus the HFD mice.

To elucidate whether the alleviating effect of ZnPA on glucolipid metabolism, hepatic steatosis and inflammation involves bile acid metabolism and HDAC3 activation, we assessed the expression levels of FXR (NR1H4) and PGC-1α in mouse liver via Western blotting. In parallel, the mRNA expression of HDAC3 in the liver and small intestine was quantified using real-time PCR. As shown in Figure 5A–D, HFD feeding expectedly suppressed FXR expression with marked decrease from 1.00 in the ND group to 0.35, representing a 65% reduction (*p* < 0.01, Figure 5A,B). However, ZnPA at 200 mg/kg significantly increased FXR expression to 0.72, a 2.06-fold elevation, compared to the HFD group (*p* < 0.05). WHB treatment could restore FXR levels to 0.81, representing a 2.31-fold increase over the HFD group (*p* < 0.01), indicating a pronounced recovery effect. As expected, treatment of ZnPA at 100 mg/kg raised FXR expression to 0.56, a 1.60-fold increase, relative to the HFD group. Similarly, the expression of PGC-1α was markedly downregulated in the HFD group with the decrease from 1.00 to 0.42, as a reduction of approximately 58% (*p* < 0.01, Figure 5A,C). Administration of ZnPA at 200 mg/kg restored PGC-1α expression to 0.76 as a 1.81-fold increase as compared to the HFD group (*p* < 0.01), while treatment of WHB at 100 mg/kg further enhanced its expression to 0.88, corresponding to a 2.10-fold increase (*p* < 0.001), and 100 mg/kg of ZnPA treatment resulted in a moderate increase to 0.60 with a 1.43-fold elevation over the HFD group.

In addition, qPCR analysis also revealed that hepatic HDAC3 expression was also suppressed by HFD feeding, decreasing from 1.00 in the ND group to 0.48, a 52% reduction (Figure 5D). H-ZnPA intervention significantly increased HDAC3 expression to 0.85, a 1.77-fold enhancement compared to the HFD group (*p* < 0.05). WHB treatment elevated HDAC3 expression to 0.76, representing a 1.58-fold increase (*p* < 0.05), while L-ZnPA treatment moderately increased it to 0.68, a 1.42-fold elevation, relative to the HFD group. Similarly, HDAC3 expression in colonic tissues was markedly reduced by HFD feeding, decreasing from 1.00 in the ND group to 0.54 with a reduction of 46% (Figure 5E). Administration ZnPA at 100 and 200 mg/kg effectively restored HDAC3 expression to 0.78 and 0.95, a 1.68- and 1.76-fold increase relative to the HFD group, respectively (*p* < 0.01), but the WHB also significantly upregulated HDAC3 expression to 0.91, a 1.68-fold increase (*p* < 0.01). Overall, 200 mg/kg of ZnPA and 100 mg/kg of WHB treatments exerted more effective regulation on the hepatic expression of FXR, PGC-1α and HDAC3 in comparison with 100 mg/kg of ZnPA.

## 4. Discussion

Obesity is not only characterized by excessive fat accumulation, but also is frequently accompanied by chronic hepatic inflammation, serving as a key driving factor in metabolic diseases such as NAFLD [36]. Here, in this study provides significant evidence that administration of zinc phytate (ZnPA), a naturally occurring organo–mineral complex derived from wheat bran (WHB), exerts potent protective effects against the HFD-induced obesity and its associated liver inflammation by regulating gut microbiota composition in mice. Mice with NAFLD often exhibit dysregulated glucose and lipid metabolism and hepatic steatosis [37]. Specifically, ZnPA treatment significantly reduced body weight gain, improved glucolipid metabolism, and alleviated hepatic steatosis (Figure 1).

The gut microbiota has been demonstrated to play a pivotal role in ameliorating obesity-associated hepatic inflammation and metabolic disorders [38]. Given that ZnPA cannot be absorbed in the upper gastrointestinal tract and is able to reach the colon, we hypothesize that ZnPA may exert its in vivo bioactivity by modulating the composition of the gut microbiota. Notably, previous study has shown that phytates are capable of altering the gut microbial composition in mice [39]. Interestingly, ZnPA also restored the HFD-caused gut microbiota dysbiosis, as evidenced by increasing alpha diversity of gut microbiota, promoting beneficial genera such as *Dubosiella* and *Ileibacterium*, and decreasing inflammation-associated taxa like the *Ruminococcus torques* group and *Coriobacteriaceae UCG-002* (Figure 2). Previous studies have also reported that probiotic supplementation improves NAFLD by increasing the abundance of *Dubosiella* and *Ileibacterium* [40]. Meanwhile, These microbial changes were associated with elevated levels of microbial metabolites, particularly inositol phosphate derivatives, SCFAs and bile acids, suggesting that ZnPA might exert its metabolic benefits through modulation of the gut ecosystem. As a result, our findings for the first time revealed a novel aspect of colon microbiota-dependent metabolic and effective mechanisms of ZnPA, as it resisted the degradation in the upper gastrointestinal tract, enabling itself to reach the colon in an intact or minimally altered form, which is consistent with previous reports [41,42]. In this study, ZnPA underwent the microbial fermentation, yielding bioactive metabolites such as inositol phosphate derivatives (InsP_6_ to InsP_3_), SCFAs (e.g., acetate, propionate, and butyrate), and bile acids (e.g., taurochenodeoxycholic acid, and deoxycholic acid). These metabolites have been reported to regulate metabolic and immune processes along the gut-liver axis [43,44,45,46].

This study is the first to investigate the colon-targeted release of inositol phosphates (InsPs) from ZnPA, including InsP_6_ (inositol hexaphosphate) and its derivatives InsP_5_, InsP_4_, and InsP_3_. The findings suggest that ZnPA reaches the colon nearly intact, where it is fermented by gut microbiota, leading to the release and accumulation of colonic inositol phosphates (InsP_3–6_). This process results in increases of 52.1% in InsP_6_, 83.3% in InsP_5_, 62.5% in InsP_4_, and 96.2% in InsP_3_. Recent studies indicate that the food-borne metabolite InsP_3_ (also known as IP3) serves as an important cellular signaling molecule, playing key roles in regulating cell growth, metabolism, and ion homeostasis [47,48,49]. Prior research demonstrated that InsP_3_ promotes the growth of human-derived intestinal organoids by stimulating HDAC3-dependent proliferation and mitigating butyrate’s inhibitory effect on colonic growth [24]. Additionally, HDAC3 regulates hepatic lipid metabolism through its deacetylation activity by inhibiting fatty acid synthesis, thereby alleviating lipid toxicity-induced liver damage [50]. In our study, 12 weeks of ZnPA treatment increased the levels of InsP_6_ and InsP_3_ metabolites (Figure 3) and significantly elevated HDAC3 expression in both the small intestine and liver (Figure 5). These results suggest a critical role for HDAC3 in mediating ZnPA’s inhibitory effects on hepatic lipid toxicity, consistent with previous research.

Bile acids are crucial regulators of lipid metabolism, particularly in cholesterol and fatty acid homeostasis. Through microbial conversion, primary bile acids are transformed into secondary bile acids (DCA and TCA), which serve as activators of the FXR pathway [51,52]. FXR, a nuclear receptor activated by bile acids, is highly expressed in the liver and intestine. It regulates genes involved in cholesterol and bile acid homeostasis, hepatic gluconeogenesis, lipogenesis, inflammation, fibrosis, and also maintains intestinal barrier integrity [53,54]. Research indicates that FXR activation increases the expression of its downstream coactivator PGC-1α, which in turn enhances hepatic fatty acid β-oxidation and mitochondrial function [55,56]. In this experiment, it was observed for the first time that after 12 weeks of ZnPA treatment, the TCA and DCA levels in the fecal metabolites of obese mice were increased (Figure 4), and activation of FXR and PGC-1αin the liver was evident (Figure 5). Notably, the trend of these results was consistent across different observations. These findings suggest that bile acids, particularly TCA and DCA as key regulatory factors in ZnPA’s ability to alleviate obesity, act as an important mediator in association with regulation and metabolism of the gut microbiota.

Short-chain fatty acids (SCFAs) are primarily produced by gut microbiota through the fermentation of dietary fibers [57]. SCFAs may mitigate lipid toxicity by modulating hepatic metabolic pathways [58]. SCFAs also act the G protein-coupled receptors (GPRs) to further suppress the NF-κB pathway, providing an additional layer of systemic anti-inflammatory regulation [59]. This study has shown that after 12 weeks of ZnPA treatment, SCFAs metabolites were increased (Figure 3), and several biochemical markers indicative of liver inflammation were elevated (Figure 1), showing significant improvement in the liver inflammation and indicating that ZnPA exerts multifaceted hepatoprotective effects by orchestrating gut microbiota composition and influencing SCFA-mediated pathways.

Based on a comprehensive analysis of three types of key metabolites as InsP_3_, bile acids and SCFAs with their associated signaling pathways, it was proposed that ZnPA enhanced hepatic expression of histone deacetylase 3, bile acid receptor FXR, and lipid metabolism coactivator PGC-1α, thereby alleviating obesity-related hepatic inflammation and metabolic disorders. Our study demonstrated that ZnPA regulated the levels of gut microbiota-derived InsP_3_, which in turn activated HDAC3, a critical deacetylase involved in fatty acid metabolism. This activation regulates lipid metabolic genes, leading to enhanced fatty acid oxidation and decreased fatty acid synthesis. Furthermore, ZnPA altered bile acid composition, thereby activating the nuclear receptor FXR, which enhanced fatty acid oxidation through its coactivator PGC-1α. Moreover, ZnPA increased the production of SCFAs, and these metabolites not only support energy metabolism but also exert anti-inflammatory effects, improving liver immune function and alleviating inflammation induced by lipid accumulation.

In conclusion, this study for the first time highlights the hepatoprotective effects of ZnPA. Such protective effect of ZnPA were primarily attributed to its regulatory effects on gut microbiota and microbial metabolism, subsequently upregulating HDAC3 to regulate fatty acid metabolism and activating FXR–PGC-1α signaling axes to regulate liver function and suppress inflammation. By challenging the conventional view of ZnPA complex (phytic acid and zinc) as inhibitor of mineral absorption, this study provides a novel perspective on the novel bioactive properties of ZnPA. These findings also offer a theoretical foundation for the rational design of ZnPA-enriched functional foods or nutraceuticals targeting metabolic diseases. However, this study has limitations. The mouse model may not fully recapitulate human physiology, and the transferability of these findings to humans remains uncertain. Moreover, the potential long-term side effects of ZnPA were not assessed. These issues should be addressed in future studies, including extended animal experiments and clinical investigations.

## Figures and Tables

**Table 1:** Effects of ZnPA on Serum Lipid Profiles in High-Fat Diet-Fed C57BL/6J Mice.

Group	ND	HFD	HFD+L-ZnPA	HFD+L-ZnPA	HFD+WHB	Normal Range
TC (mmol/L)	4.09 ± 0.46 ^b^	6.12 ± 0.60 ^a^	4.18 ± 0.40 ^b^	3.41 ± 0.53 ^b^	2.47 ± 0.34 ^c^	2.0–3.5
TG (mmol/L)	0.46 ± 0.08 ^c^	0.90 ± 0.03 ^a^	0.72 ± 0.03 ^b^	0.51 ± 0.05 ^c^	0.43 ± 0.07 ^c^	0.3–0.6
HDL (mmol/L)	0.84 ± 0.06 ^a^	0.33 ± 0.07 ^d^	0.51 ± 0.04 ^c^	0.59 ± 0.05 ^bc^	0.69 ± 0.06 ^b^	0.7–1.2
LDL (mmol/L)	0.61 ± 0.02 ^c^	0.90 ± 0.04 ^a^	0.72 ± 0.10 ^b^	0.63 ± 0.04 ^bc^	0.60 ± 0.02 ^c^	0.2–0.7

Note: Data are presented as the mean ± standard deviation (*n* = 8). Different superscript letters denote statistically significant differences (*p* < 0.05).

## Data Availability

All data generated or analyzed in this study are included in the article. Further information is available from the corresponding authors upon reasonable request.
